# Characterization of multi-drug tolerant persister cells in *Streptococcus suis*

**DOI:** 10.1186/1471-2180-14-120

**Published:** 2014-05-12

**Authors:** Jörg Willenborg, Daniela Willms, Ralph Bertram, Ralph Goethe, Peter Valentin-Weigand

**Affiliations:** 1 Institute of Microbiology, University of Veterinary Medicine, Hannover, Germany; 2 Department of Microbial Genetics, University of Tübingen, Tübingen, Germany

**Keywords:** *Streptococcus suis*, Persister cells, Multidrug tolerance, Antibiotics

## Abstract

**Background:**

Persister cells constitute a subpopulation of dormant cells within a microbial population which are genetically identical but phenotypically different to regular cells. Notably, persister cells show an elevated tolerance to antimicrobial agents. Thus, they are considered to represent a microbial ‘bet-hedging’ strategy and are of particular importance in pathogenic bacteria.

**Results:**

We studied the ability of the zoonotic pathogen *Streptococcus* (*S*.) *suis* to form multi-drug tolerant variants and identified persister cells dependent on the initial bacterial growth phase. We observed lower numbers of persisters in exponential phase cultures than in stationary growth phase populations. *S. suis* persister cells showed a high tolerance to a variety of antibiotics, and the phenotype was not inherited as tested with four passages of *S. suis* populations. Furthermore, we provide evidence that the persister phenotype is related to expression of genes involved in general metabolic pathways since we found higher numbers of persister cells in a mutant strain defective in the catabolic arginine deiminase system as compared to its parental wild type strain. Finally, we observed persister cell formation also in other *S. suis* strains and pathogenic streptococcal species.

**Conclusions:**

Taken together, this is the first study that reports multi-drug tolerant persister cells in the zoonotic pathogen *S. suis*.

## Background

Formation of persister cells by bacteria is a phenomenon that, amongst others, contributes to tolerance of a bacterial subpopulation to antimicrobial agents. Notably, this antibiotic tolerance of persister cells is distinct from genetically inherited resistance. The persister cell subpopulation has been firstly described and named nearly 70 years ago [1] and research on persister cells has identified a number of typical characteristics as debated recently [2]. Bacterial persister cells seem to represent a stage of dormancy that protects them from killing by antimicrobial substances, even in the presence of concentrations which vastly exceed the minimal inhibitory concentration (MIC). Persister cells are genetically identical to antibiotic sensitive bacteria within a population, but have a distinct phenotype in that they are tolerant to certain antibiotics [3]. Since most antibiotics target bacterial components or pathways involved in replication, the dormancy stage in persister cells is thought to be the underlying mechanism of antibiotic tolerance [4]. Nevertheless, persister celIs can switch from the dormant into a replicating stage. This ‘bet-hedging’ strategy is thought to be a survival strategy of microbial populations [5].

Two different types of persister cells have been postulated. Type I persister cells are formed in response to environmental stimuli, for instance during the initiation of the stationary growth phase, whereas type II persister cells arise stochastically within a dividing population [6,7].

A recent report proposed a ‘persistence-if-stuff-happens’ hypothesis, i.e. persister cell formation is an inevitable process due to cellular errors that produce transient states of reduced replication and/or metabolic activity in a single bacterium [8]. Nevertheless, in the last years many attempts have been made to identify molecular factors involved in the development of a persister cell subpopulation. There is increasing evidence that toxin-antitoxin modules, quorum-sensing molecules, global transcriptional regulators, and molecules of the stringent response like (p)ppGpp are involved in persister cell formation [4,9-13].

Since the first report by Bigger in 1944 [1], bacterial persister cells have been described for a number of different species, including *Escherichia coli*[14], *Staphylococcus aureus*[14,15], *Pseudomonas aeruginosa*[16], and *Mycobacterium tuberculosis*[17,18]. For most of these bacterial species persister cells have also been found in biofilms, which contribute to recalcitrant and/or recurrent infections after antibiotic therapy [4,19-25].

Little is known about persister cell formation in streptococci [9,26]. Within pathogenic streptococci, the zoonosis *Streptococcus suis* (*S. suis*) is of particular interest since it can cause very severe diseases, such as sepsis, meningitis and streptococcal toxic shock like syndrome in humans who are in close contact to pigs or pig products [27-30]. Notably, *S. suis* has been shown to be one of the most frequent causes of adult bacterial meningitis in Asian countries including Vietnam and Thailand [31,32]. *S. suis* infections are widely distributed in pigs, but can also occur in wildlife animals such as wild rabbits or wild boars [33,34]. In pigs *S. suis* is a frequent early colonizer of the upper respiratory tract. In young pigs *S. suis* is also a major cause of meningitis, arthritis, and septicemia. Thus, *S. suis* infections are a major concern in the swine producing industry as they lead to high financial losses [35].

Since antibiotics are widely used to control *S. suis* infections (in humans and in animals), we examined the ability of *S. suis* to produce antibiotic tolerant persister cells. We analyzed the effects of the initial bacterial growth phase on persister cell formation, the tolerance of these cells to different types of antibiotics, as well as persister cell levels of different *S. suis* strains and other human pathogenic streptococci. Our results show for the first time that *S. suis* forms high levels of persister cells that confer tolerance to a variety of antimicrobial compounds. We also present evidence that persister cell formation is not only found in *S. suis* but also in other streptococcal species.

## Results

### Identification of a multi-drug tolerant persister cell subpopulation in *S. suis*

**Figure 1:**
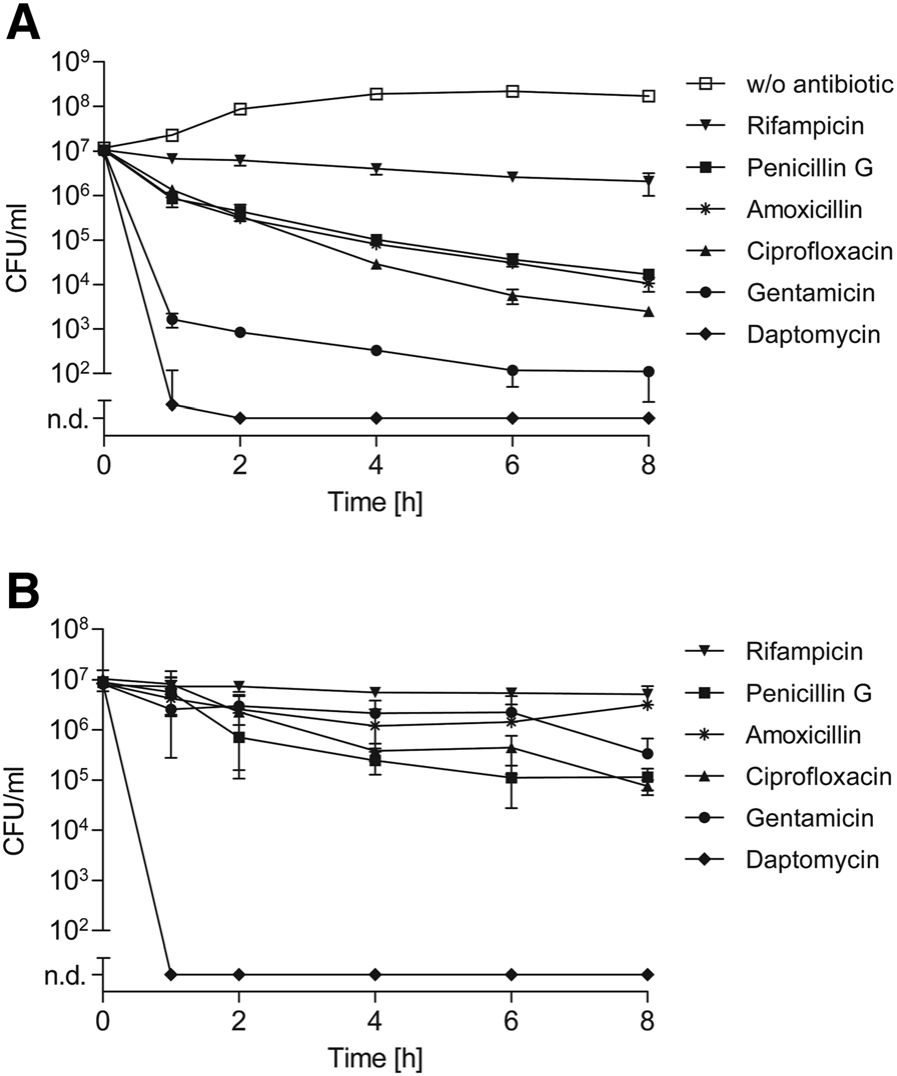
**Killing kinetics of *S. suis *exposed to different antibiotics.** Exponential **(A)** or stationary **(B)** grown *S. suis* strain 10 was treated with 100-fold MIC of indicated antibiotics over time. The limit of detection was defined as 100 CFU/ml throughout all killing experiments. All lower bacterial numbers were considered as not detectable (n. d.). The values are means of two biological replicates and error bars indicate the standard deviation. An untreated culture without any antibiotic challenge (w/o antibiotic) served as a control.

Since persister cell formation is important for antibiotic tolerance and recurrent infections, we studied the occurrence of persister cells in the zoonotic pathogen *S. suis* using a highly virulent serotype 2 strain, strain 10. First we determined the minimal inhibitory concentration (MIC) of six antibiotics with different modes of action for exponential grown *S. suis* strain 10 by the standard microdilution assay (see Additional file 1: Table S1), because one main characteristic of persister cells is the ability to tolerate concentrations of different antimicrobial compounds above the MIC. Following, to test whether *S. suis* is capable of producing persister cells that tolerate antibiotic treatment, we performed antibiotic killing experiments with a 100-fold MIC of each antimicrobial compound. Antibiotic challenge was performed with cultures grown either to exponential or stationary phase. Since a 100-fold MIC should inactivate antibiotic-sensitive normal growing bacteria, we assumed that this treatment would result in characteristic biphasic-killing characterized by an initial rapid killing of the bulk of the bacterial population followed by a distinct plateau of surviving drug tolerant persister cells [6]. As depicted in Figure 1A, gentamicin treatment of exponential grown *S. suis* resulted in decrease of bacterial CFU by three orders of magnitude within the first hour and a subsequent plateau phase in the following hours. When we applied β-lactam antibiotics and ciprofloxacin the killing was not as pronounced as observed for gentamicin, nevertheless a slow decrease of life counts was seen over time. Nearly no killing was observed after treatment with rifampicin. In contrast, daptomycin was able to completely kill the bacterial population without detectable survival of persister cells. These data indicate that within an exponential grown *S. suis* culture a subpopulation of antibiotic tolerant persister cells exists, which show different degrees of tolerance depending on the class of antibiotic.

Next we studied the persister cell levels of stationary grown *S. suis* since for several other bacterial species a drastic increase in persister levels has been reported at the onset of stationary growth phase [4]. Antibiotic treatment of stationary cultures of *S. suis* with 100-fold MIC resulted in a substantial drug tolerance, i.e. a distinct biphasic killing pattern such as seen with exponential cultures was not observed (Figure 1A vs. B). Only a slight decrease in numbers of CFU was detected over time after treatment with β-lactams, ciprofloxacin and gentamicin. In the case of gentamicin a relative difference of approximately three logarithmic orders in CFU was recorded after the first hour of antibiotic treatment, when comparing populations of exponential and stationary grown *S. suis*. Notably, growth to the stationary growth phase did not enhance the tolerance of *S. suis* to the cyclic lipopeptide daptomycin which completely killed the *S. suis* population after only one hour of treatment. Taken together, the killing kinetics revealed that under the conditions tested *S. suis* develops a growth phase dependent subpopulation showing antibiotic tolerance to a variety of antimicrobial compounds except daptomycin.

### The persister cell phenotype of *S. suis* is not inherited and dominated by type I persisters

**Figure 2:**
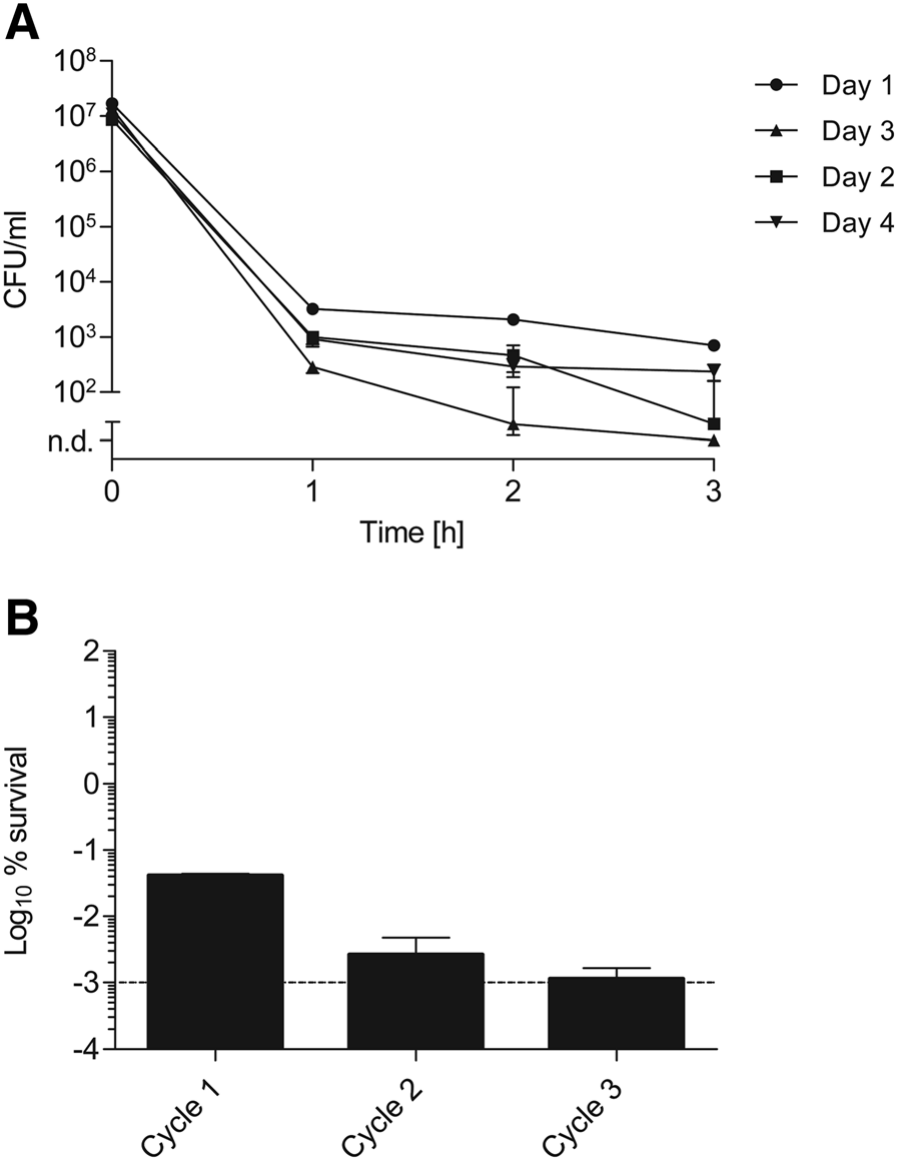
**Test for the heritability of persistence and elimination of persister cells. (A)** Exponential grown *S. suis* strain 10 was treated with 100-fold MIC of gentamicin for three hours, and at indicated time points CFU were determined. Subsequently, surviving bacteria were incubated in fresh THB media overnight, then grown to early logarithmic phase and challenged with 100-fold MIC of gentamicin. This procedure was repeated for four consecutive cycles. The values are means of three biological replicates and error bars indicate the standard deviation. **(B)***S. suis* strain 10 was sequentially grown to early exponential growth phase. At each cycle CFU of the initial inoculum and of surviving bacteria after a one-hour 100-fold MIC gentamicin challenge were determined. Data were expressed for each cycle as percentage of surviving bacteria in relation to the initial inoculum before antibiotic treatment. The dotted line represents the limit of detection. Standard deviation is shown for three replicates.

In contrast to genetically encoded antimicrobial resistance, multidrug tolerance of persister cells is a transient and non-heritable phenotype [10,26]. To test heritability of antimicrobial tolerance, exponential grown *S. suis* was treated with 100-fold MIC of gentamicin and the surviving population was used to repeat a new cycle. Four consecutive cycles were tested. Gentamicin was selected for these experiments since this treatment resulted in pronounced biphasic killing curves in the first hours after antibiotic treatment. As depicted in Figure 2A, tolerance to gentamicin of the initial population was not transferred to following *S. suis* generations. The characteristic biphasic killing curve upon antibiotic treatment was observed irrespective of the number of passages. These results suggest that the formation of a *S. suis* persister cell subpopulation and antimicrobial tolerance is not inherited and of transient nature.

In order to dissect whether type I or type II persisters are responsible for gentamicin tolerance, we performed a persister cell elimination assay. In this assay the formation of type I persisters is suppressed by sequential re-inoculation of an early exponential culture. This procedure leads to dilution of type I persisters whilst stochastically build type II persister cell levels should remain constant. As depicted in Figure 2B the percentage of antibiotic tolerant persisters decreased sequentially after 100-fold MIC gentamicin challenge when the bacterial culture was kept in the early growth phase for three cycles. This data indicate that gentamicin tolerant persisters are not or only rarely produced in the early exponential growth phase and that most of the tolerant bacteria represented type I persisters. These were probably ‘left overs’ from the overnight culture and became diluted within repeated cycles of exponential growth.

### *S. suis* persister cells also tolerate combinations of different antibiotics

**Figure 3:**
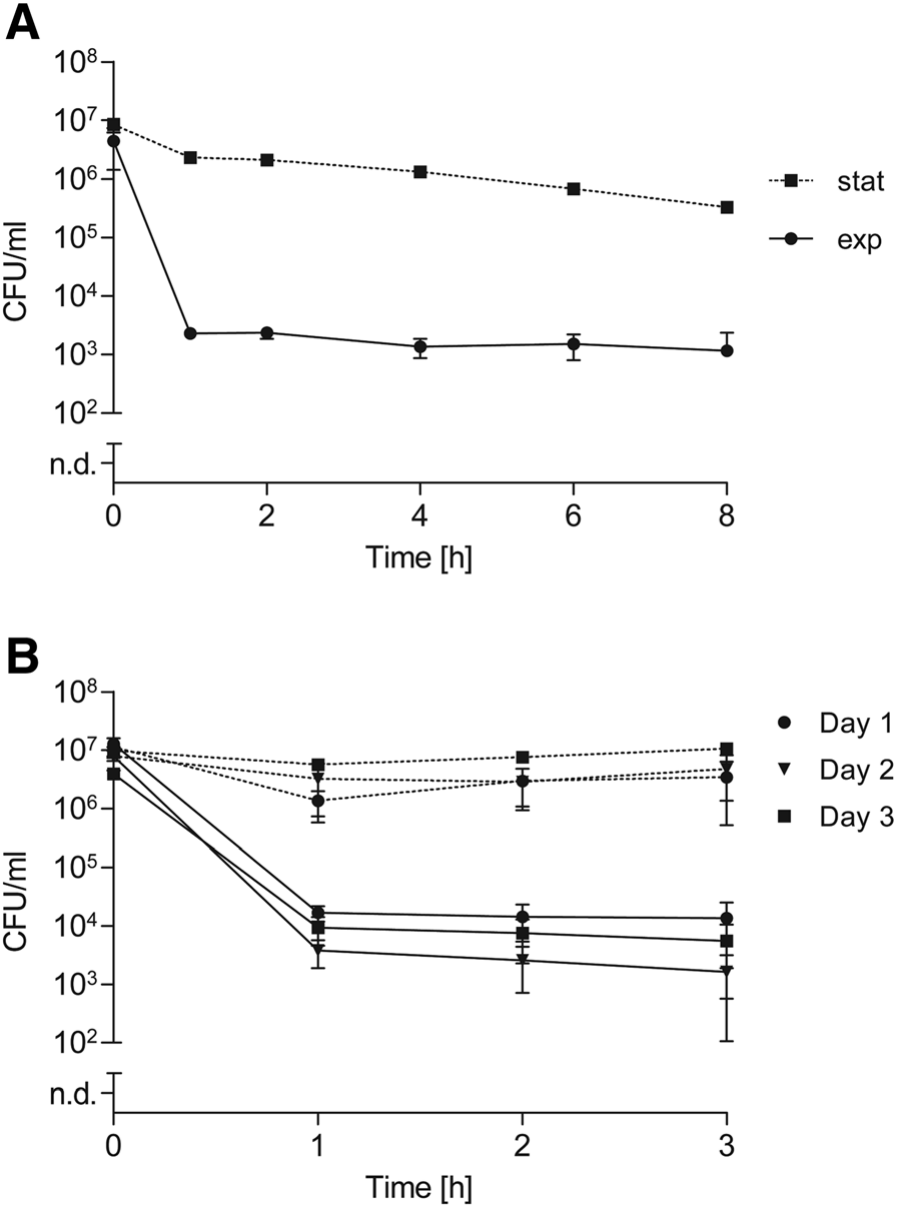
**Time-dependent killing after combined antibiotic treatment. (A)** Exponential (solid line) and stationary (dotted line) grown *S. suis* strain 10 was exposed to a combined antibiotic treatment of 200-fold MIC of penicillin and 4-fold MIC of gentamicin over time. **(B)** This penicillin/gentamicin combination was also used in a heritability test with exponential (solid line) and stationary (dotted line) grown *S. suis* in three consecutive cycles. The values are means of two biological replicates plated in triplicate. Error bars indicate the standard deviation.

Antibiotics like penicillin are frequently used to treat *S. suis* infections, sometimes in combination with other antibiotics like aminoglycosides. However, relapses of *S. suis* infections in pigs and humans have been reported [36]. Furthermore, penicillin and gentamicin are widely used in standard antibiotic protection assays to quantify intracellular bacteria in *in vitro* cell culture experiments. Therefore, we investigated *S. suis* tolerance against a combination of penicillin (200-fold MIC) and gentamicin (4-fold MIC) that correspond to the concentrations applied in these antibiotic protection experiments. After simultaneous treatment of exponential grown *S. suis* with penicillin and gentamicin we observed a biphasic killing curve characterized by a rapid decrease of CFU numbers within the first hour and a subsequent plateau of surviving bacteria persisting for more than 8 hours (Figure 3A). The killing kinetics of stationary grown bacteria treated similarly resembled treatment with gentamicin alone, as depicted in Figure 1B. Similar to what we observed after treatment with a single antibiotic, the tolerance to a combination of penicillin and gentamicin was not inherited, as revealed from heritability tests (Figure 3B). These data suggest that *S. suis* persister cells are capable of tolerating not only single antibiotics, but also a combination of penicillin and aminoglycosides.

### Persister cell formation in *S. suis* is affected by the global transcriptional regulator CcpA and the catabolic arginine deiminase system

**Figure 4:**
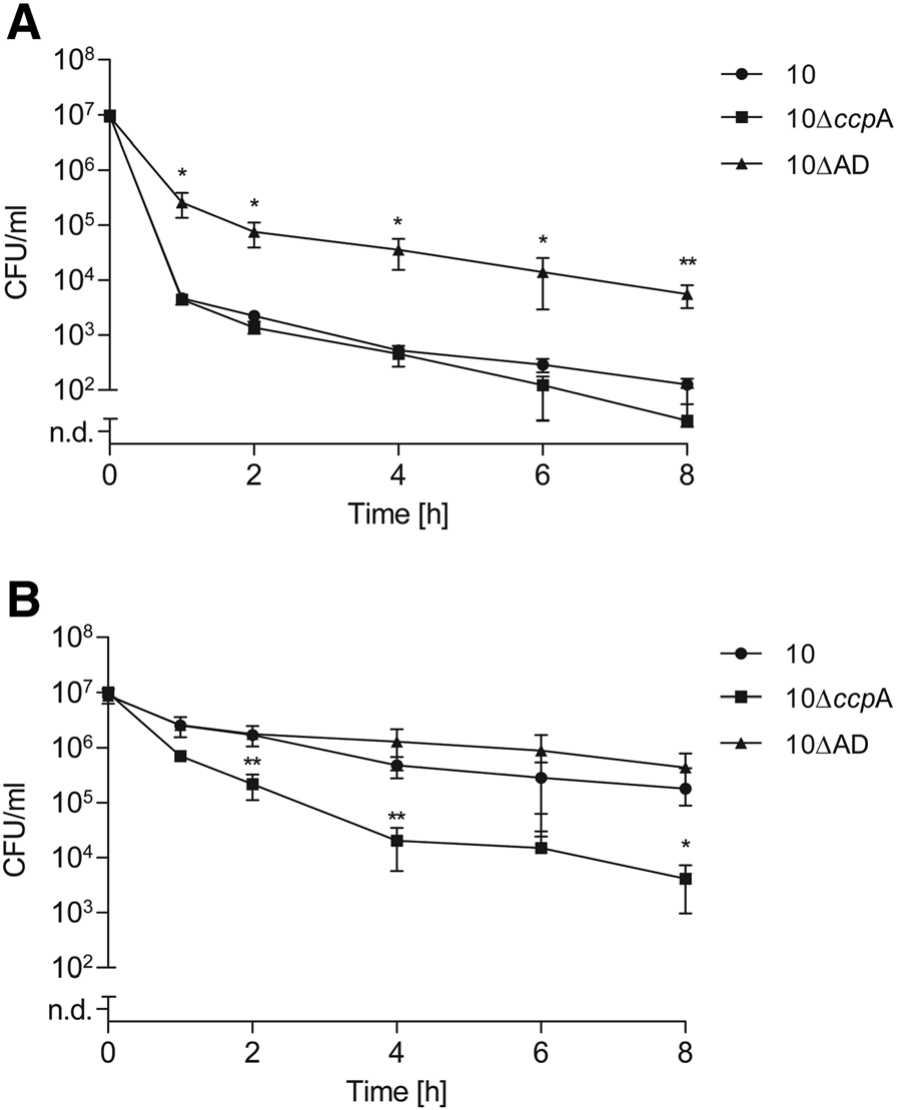
**Effect of specific gene inactivation on *S. suis *persister formation.** Exponential **(A)** or stationary **(B)** grown *S. suis* strains were treated with 100-fold MIC of gentamicin over time. Persister cell levels were determined for the wild type strain 10, and its knock-out mutant strains 10∆*ccp*A and 10∆AD, which lack the genes coding for the global transcriptional regulator CcpA and the catabolic arginine deiminase system, respectively. The values are means of three biological replicates and error bars indicate the standard deviation. Significant differences to wildtype persister levels were calculated by a one-tailed *t*-test (*, *P* < 0.05; **, *P* < 0.01).

Several genes and gene products have been implicated in antibiotic tolerance, like toxin-antitoxin (TA) modules, quorum-sensing molecules and global transcriptional regulators [4,9]. In previous studies we have shown that CcpA is a pleiotropic regulator of *S. suis* carbon metabolism, virulence gene expression and the expression of the arginine deiminase (AD) system [37-39]. The latter is crucial for bacterial survival in acidic environments and is most likely required for alternative ATP generation. Hence, we tested respective *S. suis* mutant strains 10Δ*ccp*A and 10ΔAD for gentamicin tolerant persister cells. CFU of bacterial strains grown to the exponential growth phase were determined over time after treatment with 100-fold MIC gentamicin. The gentamicin MIC values of the mutant strains did not differ from those of the wild type strain. No change in persister levels was observed for exponential grown strain 10Δ*ccp*A, whereas the AD mutant strain 10ΔAD showed an approximately two log-fold higher persister cell level over time compared to the wild type (Figure 4A). This difference was abrogated when stationary growth phase cultures were challenged by gentamicin (Figure 4B). Interestingly, during the later growth phase the persister level of strain 10Δ*ccp*A decreased as compared to the wild type and strain 10ΔAD.

### Persister cell formation occurs in different *S. suis* strains and streptococcal species

**Figure 5:**
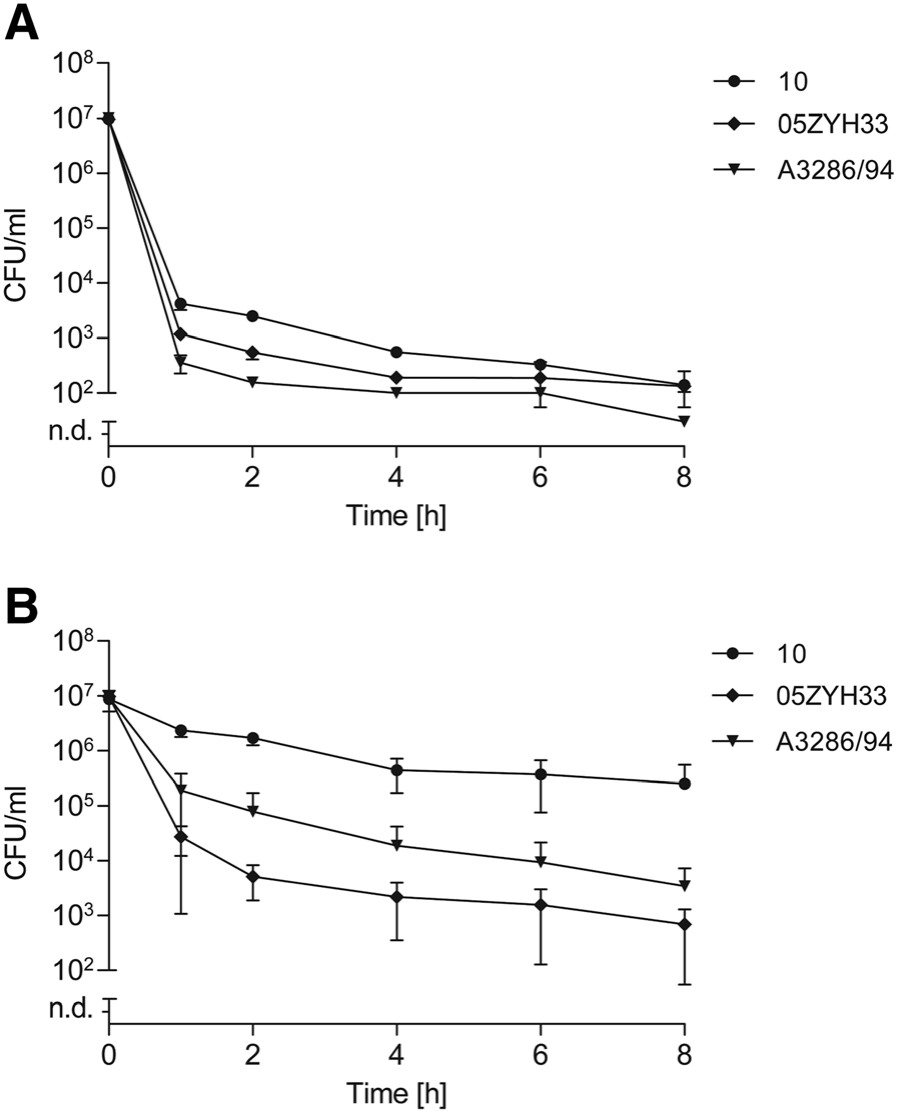
**Persister cell levels of different *S. suis *strains.** Exponential **(A)** or stationary **(B)** grown *S. suis* strains were treated with 100-fold MIC of gentamicin over time. Persister cell levels were determined for the porcine serotype 2 isolate strain 10, a porcine serotype 9 isolate strain A3286/94, and a human serotype 2 isolate strain 05ZYH33. The values are means of two biological replicates and error bars indicate the standard deviation.

Next, we tested antibiotic tolerance and persister cell formation in other *S. suis* strains and streptococcal species. For this, we analyzed a human serotype 2 isolate (strain 05ZYH33) originating from a *S. suis* outbreak in China and a serotype 9 strain (strain A3286/94) isolated from a pig with meningitis [40,41]. The MIC values of gentamicin for strain 05ZYH33 and strain A3286/94 are given in Additional file 1: Table S1. In all strains, treatment with 100-fold MIC of gentamicin induced the characteristic biphasic killing curve and resulted in a complete killing of bacteria after 24 hours. No substantial differences could be observed between strains in the exponential growth phase (Figure 5). On the other hand, using stationary cultures strain 10 showed the highest degree of drug tolerance. Strains A3286/94 and 05ZYH33 were killed more efficiently, especially during the first hour of antibiotic treatment, with persister cell differences of up to two log-fold CFU. After 24 hours of gentamicin treatment viable bacteria could only be recovered in strain 10, but not in strains A3286/94 and 05ZYH33, respectively (data not shown). These data indicate that various *S. suis* strains and serotypes form persisters with different frequencies and antibiotic tolerance characteristics.

**Figure 6:**
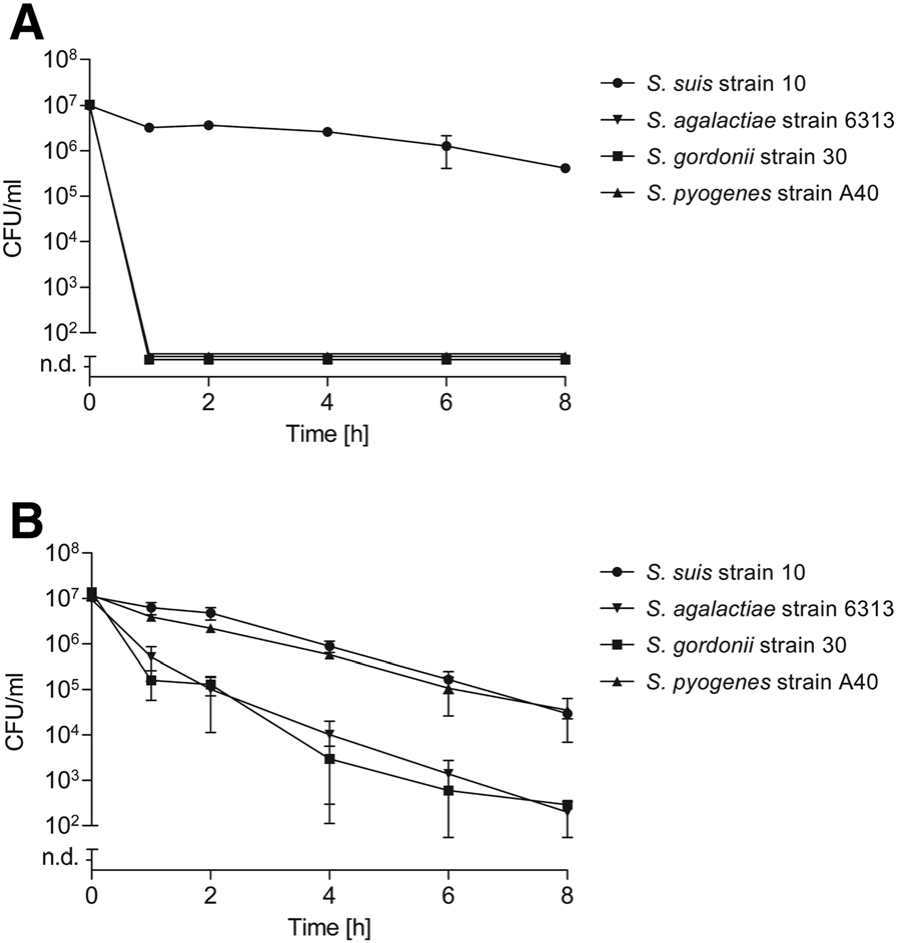
**Persister cell levels of selected human pathogenic streptococci.** Overnight cultures of indicated streptococcal strains were treated with 100-fold MIC of gentamicin **(A)** or 100-fold MIC of ciprofloxacin **(B)** over time. The values are means of two biological replicates and error bars indicate the standard deviation.

Since antibiotic tolerance has been reported for other streptococcal species [42-44] we studied persister cell formation in selected strains of other streptococci, including *S. pyogenes*, *S. agalactiae*, and *S. gordonii* after treatment with 100-fold MIC gentamicin. The determined MIC values for each strain are listed in Additional file 1: Table S1. Interestingly, in contrast to *S. suis* neither exponential nor stationary grown streptococci of the tested strains displayed a gentamicin tolerant subpopulation (data not shown). Notably, we could not detect any gentamicin tolerant subpopulation for *S. pyogenes*, *S. gordonii*, and *S. agalactiae* overnight cultures as shown in Figure 6A. On the other hand, treatment with 100-fold MIC of ciprofloxacin resulted in a drug-specific tolerance for at least 8 hours (Figure 6B). The proportion of ciprofloxacin tolerant bacteria was higher for *S. suis* strain 10 and *S. pyogenes* strain A40 as compared to the other streptococcal species. These data indicate that drug tolerant subpopulations might also occur in other streptococcal species, but the extent of tolerance seems to vary between different antibiotics.

## Discussion

Generation of bacterial persister cells is important not only with respect to the understanding of population dynamics but also concerning antibiotic tolerance in respective therapy of infections [45]. Accordingly, there is growing evidence that bacterial persisters are involved in relapses of refractory bacterial infections and in the establishment of resistance mechanisms in bacteria [21]. Owing to this it seems not surprising that persister cells have been described for numerous pathogenic bacteria. In this study we have shown for the first time that *S. suis* forms multi-drug tolerant persister cells. Even though 100-fold MIC is unlikely to be achieved in therapy of natural infections, we assumed that this treatment would be a method of choice to identify highly drug-tolerant persister cells of *S. suis* in accordance to results reported for *S. aureus*[15]. By this we identified persister cell formation in three different *S. suis* strains, suggesting that this phenomenon may be a general trait among this species. Though this has to be further confirmed by testing more *S. suis* strains and antibiotics that are of higher clinical relevance to treat *S. suis* infections in pigs and humans, persister cells should be considered in the future in cases of ineffective antibiotic treatments or when studying antibiotic tolerance of *S. suis*.

In line with several previous studies [3,14,22,46] the number of persisters observed was higher during stationary growth of *S. suis* when compared to exponential grown bacteria. Type I persisters were found to be the main source of antibiotic tolerance in our experiments. Among other stress signals, nutrient limitation in stationary growth is thought to be a trigger inducing down-regulation of the metabolic activity and bacterial dormancy in energy-deprived cells which can protect the bacteria from antibiotic killing. We found some hints for involvement of the catabolic enzyme system ADS, since approximately two log-fold higher levels of persister cells were found in the exponential growth phase of an arginine deiminase knock-out strain (10ΔAD) as compared to its wild type strain. In *S. suis* the arginine deiminase system metabolizes arginine as a substrate to produce energy in form of ATP [38]. The diminished ATP levels may lead to reduced general metabolic activity of strain 10ΔAD that might explain the slower growth rate (see Additional file 2: Figure S1) and enhanced number of antibiotic tolerant persister cells. Furthermore, the *ccp*A deficient strain exhibited lower numbers of persister cells in the stationary growth phase when compared to the wild type. This is in agreement with studies in *S. gordonii* showing that a *ccp*A knock-out resulted in an increased sensitivity of the bacteria to penicillin treatment [47]. Since CcpA is a pleiotropic regulator that is important for a balanced metabolic flux in the central carbon metabolism, the alteration of central metabolic processes may influence persister cell formation of *S. suis*. Accordingly, an interplay between carbohydrate consumption and formation of persisters has recently been demonstrated for *E. coli*[12]. Further studies are needed to clarify the mechanisms involved in CcpA and/or arginine deiminase dependent changes in antibiotic tolerance of *S. suis*.

When using antibiotics with varying modes of action, resulting killing profiles were quite different, ranging from pronounced biphasic killing patterns to nearly plane curves, at least for exponential grown *S. suis*. These findings seem to be highly dependent on the type of antibiotic used, which is also emphasized by the fact that treatment with the β-lactam antibiotics amoxicillin and penicillin resulted in similar killing curves. Thus we speculated a common mechanism of tolerance for certain antibiotics and tested the gentamicin tolerance in other streptococcal species. *S. suis* strain 10 highly tolerated 100-fold MIC of gentamicin, whereas the other streptococcal strains were completely killed after one hour. These data suggest that a specific mechanism for gentamicin tolerance of *S. suis* persisters may have evolved and that this is, most likely, not due to a shared genetic background within the genus *Streptococcus*. Interestingly, after gentamicin treatment of *S. suis* we also observed a small-colony-variant (SCV) like phenotype (data not shown) that has also been reported for *S. aureus* upon aminoglycoside treatment [15,48]. Although it reverted to the typical large-colony phenotype after subcultivation, it remains to be elucidated if this phenotype will change to a stable phenotype after longer exposure times and altered antibiotic tolerance to aminoglycosides. However, at the stationary growth phase the investigated *S. suis* strain 10 highly tolerated several antimicrobials targeting different bacterial components over time. Given the high rate of multi-drug tolerant cells produced by *S. suis* strain 10 during stationary growth, it was remarkable that the cyclic lipopeptide daptomycin efficiently eradicated this subpopulation. This is in contrast to observations that in *S. aureus* 100-fold MIC of daptomycin failed to eradicate stationary phase cultures [15]. Even though the MIC for daptomycin is rather high when compared to that of other streptococcal species [49] this treatment eradicated *S. suis* persister cells *in vitro*.

In the last years bacterial persistence and enhanced antibiotic tolerance was intensively discussed in the context of recurrent infections caused by bacterial pathogens. Interestingly, a human case of recurrent septic shock due to a *S. suis* serotype 2 infection has previously been reported [50]. Together with our present study this suggests a clinical relevance of *S. suis* persisters. Although experimental evidence for *S. suis* persister cell and biofilm formation *in vivo* is yet missing, *S. suis* is able to produce biofilms *in vitro* that tolerate antibiotic challenge [51,52]. Given the fact that the *S. suis* colonization rate of pigs is nearly 100% [35,53,54] and that antibiotic treatment with penicillin, ampicillin, or ceftiofur failed to eliminate the tonsillar carrier state of *S. suis* in swine [55], it is plausible to speculate that persister cells, possibly also as part of biofilm structures, may contribute to the observed problems in antibiotic treatments. Indeed, *P. aeruginosa* persister cells have been described as the dominant population responsible for drug tolerance in biofilms [22].

## Conclusions

Our study showed that the zoonotic pathogen *S. suis* is able to form a multi-drug tolerant persister cell subpopulation. *S. suis* persister cells tolerated a variety of antimicrobial compounds that were applied at 100-fold of MIC and could be detected in different *S. suis* strains. Thus, our study provides a basis for future studies on the role of *S. suis* persister cells in bacterial colonization of host tissues, general antibiotic tolerance, and recurrent infections.

## Methods

### Bacterial strains, media, and growth conditions

All bacterial strains investigated in this study (listed in Table 1) were grown in complex Todd Hewitt Broth (THB, Becton Dickinson Diagnostics) medium at 37°C. If not stated otherwise cryo-conserved bacterial stocks were used in the experiments. Preliminary experiments with cryo-conserved and freshly prepared bacterial cultures had revealed no significant differences in persister cell formation assays (data not shown), similar to what has been reported for *E. coli*[6]. For the preparation of bacterial stocks, overnight cultures were diluted to an optical density at 600 nm (OD_600_) of 0.02 in fresh THB medium and further incubated until bacteria reached either the early exponential (exp) or stationary (stat) growth phase as depicted in Additional file 2: Figure S1. Then 19 ml of exponential grown or 4 ml of stationary grown bacterial cultures were collected and centrifuged at 4000 × *g* for 10 min at 4°C. Bacterial pellets were washed once in phosphate-buffered saline, resuspended in THB medium containing 15% glycerol (v/v), and aliquots were immediately shock frozen in liquid nitrogen. Frozen cultures were kept at −80°C until use and numbers of viable cells were determined by serial plating on sheep blood Columbia agar plates. All antibiotic treatments were performed in chemically defined medium, RPMI 1640 (Life Technologies), which is routinely used in cell culture.

**Table 1:** Bacterial strains used in this study

**Strain**	**Description**	**Reference**
*S. suis*		
10	Virulent serotype 2 strain, porcine isolate	[56]
10Δ*ccp*A	Strain 10 *ccp*A mutant; *ccp*A::Em^R^	[39]
10ΔAD	Strain 10 arginine deiminase operon mutant; *arcA*::Spc^R^	[38]
05ZYH33	Virulent serotype 2 strain, isolate from human outbreak in China	[40]
A3286/94	Virulent serotype 9 strain, porcine isolate	[41]
*S. agalactiae*		
6313	A clinical isolate belonging to serotype III	[57]
*S. gordonii*		
30		[58]
*S. pyogenes*		
A40	A clinical isolate belonging to M type 12	[59]

### Antibiotics and determination of minimal inhibitory concentration (MIC)

Daptomycin (commercial Cubicin®) analytic grade powder was purchased from Novartis Pharma. Penicillin G, ciprofloxacin, amoxicillin, and rifampicin were purchased from Sigma, and gentamicin from Roth. The antimicrobial solutions were prepared freshly prior to each application according to the manufacturers’ recommendations.

The MIC of each antibiotic was determined in duplicate by the microdilution technique in 96-well plates. Serial two-fold dilutions of different antibiotics prepared in RPMI 1640 medium were inoculated each with 5 × 10^5^ colony forming units (CFU) of exponential grown cryo-conserved bacteria per well. MICs were also determined for freshly prepared bacterial cultures and did not significantly differ from MICs of cryo-conserved bacteria (data not shown). Plates were covered with a Breathe-Easy® sealing membrane to avoid evaporation and incubated for 24 hours at 37°C. The lowest antibiotic concentration that inhibited visible bacterial growth was defined the MIC. The determined MIC values are listed in Additional file 1: Table S1.

### Test for persister cell formation

Chemically defined RPMI 1640 medium was inoculated with 1 × 10^7^ CFU of either exponential or stationary grown cryo-conserved bacteria. Freshly prepared antimicrobial substances were added at a final concentration of 100-fold MIC, if not stated otherwise. Suspensions were incubated with end-over-end rotation at 37°C. Samples were taken after 1, 2, 4, 6, and 8 hours for determination of CFU by serial dilution and plating. For this 100 μl of bacterial suspensions were immediately harvested by centrifugation, once washed in sterile 0.85% NaCl solution and spotted as 10 μl aliquots on sheep blood Columbia agar plates in serial dilutions. Plating of the aliquots was performed in triplicates and all antibiotic killing experiments were performed at least with two biological replicates. Bacterial colonies were counted 24 and 48 hours after incubation at 37°C to ensure detection of slow growing bacteria. The results were analyzed with the GraphPad Prism 5 software and expressed in CFU/ml on a logarithmic scale. The limit of detection was defined as 100 CFU/ml and lower bacterial numbers were considered not detectable (n. d.). If indicated statistical significance was determined by one-sided Student *t* test.

### Heritability of persistence

An overnight culture was diluted to an OD_600_ of 0.02 in fresh THB medium and further incubated until the early exponential growth phase was reached. Then bacteria were harvested by centrifugation, once washed with PBS, and inoculated in fresh RPMI medium containing 100-fold MIC of the respective antibiotic to a final bacterial concentration of 1 × 10^7^ CFU/ml. The suspensions were incubated at 37°C with moderate end-over-end rotation. Samples were taken hourly as indicated and the CFUs were determined after removal of remaining antibiotics by washings as described above. After 3 hours of antibiotic treatment (surviving) bacteria were collected by centrifugation, once washed in PBS, inoculated in fresh THB medium and grown overnight. This culture was then used to start a new cycle of antibiotic treatment with exponential grown bacteria. This procedure was repeated with three consecutive cycles and the experiment performed at least with two biological replicates. Colonies were counted and CFUs calculated as described above.

### Test for persister cell elimination

To dissect whether the antibiotic tolerant persister population of *S. suis* strain 10 comprises type I or type II persister cells, we performed a persister cell elimination test as described by Keren *et al.*[14], with some modifications. Briefly, an overnight culture of *S. suis* strain 10 was adjusted to OD_600_ = 0.02 in fresh THB medium and further incubated until bacteria reached OD_600_ = 0.2. Then, aliquots of this culture were used to inoculate fresh THB medium to OD_600_ = 0.02 for a further cycle and to determine persister cell levels after a 100-fold MIC gentamicin challenge. A gentamicin challenge was done as described for the test of heritability of persistence with the exception that the antibiotic treatment lasted one hour. Dilution-growth cycles with subsequent antibiotic challenge were repeated thrice. For each cycle the initial inoculum before and the surviving bacteria after antibiotic challenge were determined by CFU counting. Data were expressed as percentage of surviving bacteria in relation to the initial inoculum before antibiotic treatment.

## Competing interests

The authors declare that they have no competing interests.

## Authors’ contributions

JW and DW carried out the experiments and analyzed the data. RB helped with the design of the study and draft of the manuscript. JW, RG and PVW conceived the study, participated in its design and coordination and helped to draft the manuscript. All authors read and approved the final manuscript.

## Supplementary Material

Additional file 1: Table S1 MIC values of antimicrobial compounds (μg/ml) for different streptococcal strains. ND stands for ‘not determined’. Click here for file

Additional file 2: Figure S1 Growth kinetics of selected *S. suis* strains, isogenic mutants of *S. suis* strain 10, and strains of other streptococcal species in THB medium. For antibiotic tolerance assays bacteria were grown in complex THB medium and harvested at an OD_600nm_ of 0.2, reflecting the early exponential growth phase, or at the stationary growth phase of each strain that is indicated by a red coloured symbol in the graph. (A) Growth curves of selected *S. suis* strains and isogenic mutants of *S. suis* strain 10. (B) Growth curves of selected strains of other streptococcal species. Click here for file
