# Supplementary material for: Characterization of multi-drug tolerant persister cells in Streptococcus suis
Source: BMC Microbiol. 2014 May 12;14:120. doi: 10.1186/1471-2180-14-120 (PMC4040513; doi:10.1186/1471-2180-14-120)
Supplement: Additional file 1: Table S1 — MIC values of antimicrobial compounds (μg/ml) for different streptococcal strains. ND stands for ‘not determined’. [file 1471-2180-14-120-S1.pdf]

Additional file 1:Table S1. MIC values of antimicrobial compounds (µg/ml) for different streptococcal strains. ND stands for ´not determined´.

**Bacterial strain**	Gentamicin (aminoglycoside)	Penicillin G (β-lactam)	Amoxicillin (β-lactam)	Ciprofloxacin (fluoroquinolone)	Rifampicin (rifamycin)	Daptomycin (cyclic lipopeptide)
Streptococcus suis						
10	25.00	0.05	6.25	1.56	3.12	3.12
10Δ*ccpA*	25.00	ND	ND	ND	ND	ND
10ΔAD	25.00	ND	ND	ND	ND	ND
A3286/94	25.00	ND	ND	ND	ND	ND
05ZYH33	50.00	ND	ND	ND	ND	ND
Streptococcus gordonii						
30	3.12	ND	ND	1.56	ND	ND
Streptococcus pyogenes						
A40	6.25	ND	ND	1.56	ND	ND
Streptococcus agalactiae						
6313	25.00	ND	ND	6.25	ND	ND
