# Supplementary figures and images for: Characterization of multi-drug tolerant persister cells in Streptococcus suis
Source: BMC Microbiol. 2014 May 12;14:120. doi: 10.1186/1471-2180-14-120 (PMC4040513; doi:10.1186/1471-2180-14-120)

**A**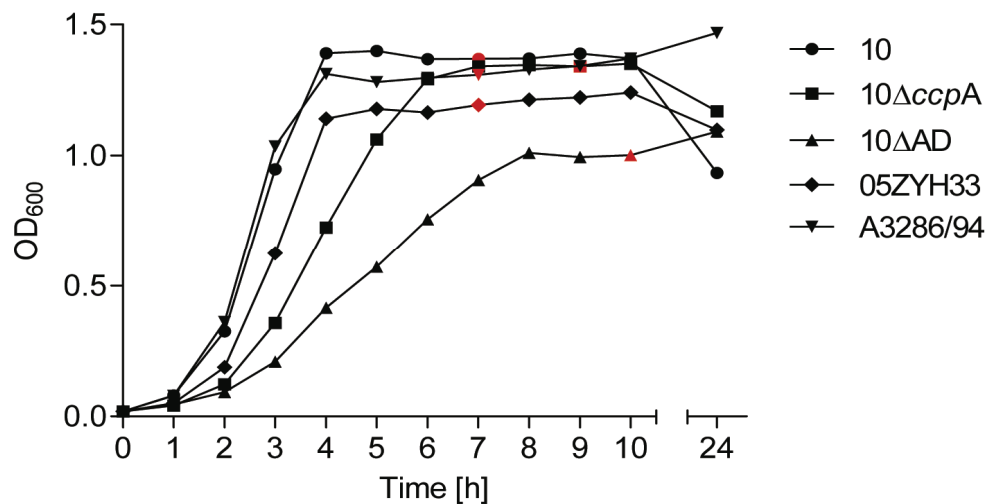**B**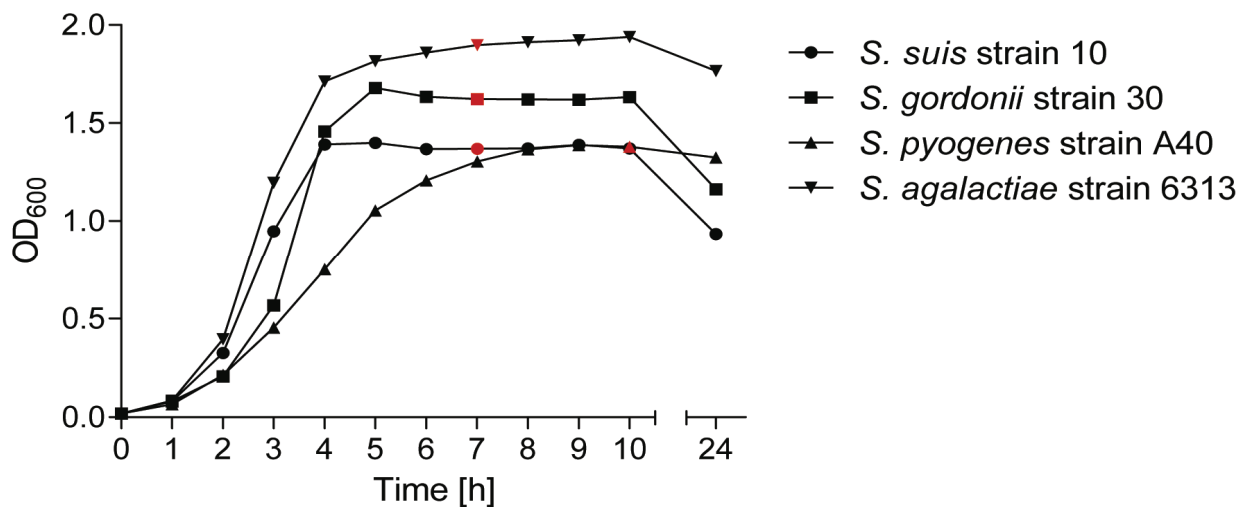

Supplement: Additional file 2: Figure S1 — Growth kinetics of selected S. suis strains, isogenic mutants of S. suis strain 10, and strains of other streptococcal species in THB medium. For antibiotic tolerance assays bacteria were grown in complex THB medium and harvested at an OD600nm of 0.2, reflecting the early exponential growth phase, or at the stationary growth phase of each strain that is indicated by a red coloured symbol in the graph. (A) Growth curves of selected S. suis strains and isogenic mutants of S. suis strain 10. (B) Growth curves of selected strains of other streptococcal species. [file 1471-2180-14-120-S2.pdf]
